# Influence of Mo on the Microstructure and Corrosion Behavior of Laser Cladding FeCoCrNi High-Entropy Alloy Coatings

**DOI:** 10.3390/e24040539

**Published:** 2022-04-12

**Authors:** Wenjuan Li, Wenmin Guo, Hongling Zhang, Huanhuan Xu, Liang Chen, Junshan Zeng, Bin Liu, Zhibing Ding

**Affiliations:** 1 College of Mechanical and Energy Engineering, Shaoyang University, Shaoyang 422000, China; liwenjuan101@gmail.com (W.L.); zhanghonglin3714@gmail.com (H.Z.); xuhuanhuan133@gmail.com (H.X.); chenliang1566@gmail.com (L.C.); zengjunshan180@gmail.com (J.Z.); 3952@hnsyu.edu.cn (Z.D.); 2 Key Laboratory of Hunan Province for Efficient Power System and Intelligent Manufacturing, Shaoyang University, Shaoyang 422000, China; 3 State Key Lab of Powder Metallurgy, Central South University, Changsha 410083, China

**Keywords:** high entropy alloy, coating, laser cladding, corrosion, microstructure

## Abstract

FeCoCrNi and FeCoNiCrMo_0.2_ high-entropy alloy powders were prepared by gas atomization. Two kinds of coatings were prepared on the surface of 304 stainless steel by laser cladding technology. The effect of Mo element on the microstructure of laser cladding FeCoCrNi coating and its corrosion behavior in 3.5 wt.% NaCl solution was investigated. Both FeCoCrNi and FeCoCrNiMo_0.2_ powders exhibit a single-phase FCC structure. Due to the remelting and multiple heat treatments during the preparation of the laser cladding coating, a small amount of σ and μ phases appeared in the FeCoCrNiMo_0.2_ coating. The microstructures of the two coatings from the bonding area to the top layer are planar, columnar and equiaxed grains, respectively. The addition of the Mo element causes the dendrite size in the middle region of the FeCoCrNiMo_0.2_ coating increases significantly and exhibits obvious orientation characteristics. FeCoCrNiMo_0.2_ coating has high corrosion potential (−0.01 *V*_SHE_) and low current density (0.94 × 10^−7^ A/cm^2^) in 3.5 wt.% NaCl solution, showing excellent corrosion resistance. The passivation film formed on corroded the FeCoCrNiMo_0.2_ coating contains high content of oxides of Cr and Mo. The addition of the Mo element enhances the compactness and pitting resistance of the passivation film.

## 1. Introduction

During the exploitation of marine resources, large-scale marine engineering equipment such as ships, underwater manipulators and seabed exploration equipment are exposed to complex marine environments for a long time [1]. The strength, toughness, low-temperature toughness and corrosion resistance of structural materials are important factors affecting their service life. The strength and corrosion resistance of traditional stainless steel is limited, and it is gradually difficult to meet the needs of engineering equipment in extreme marine environments [2].

In the past two decades, the concept of high-entropy alloy has provided new alloy design strategies and has become a new research hotspot [3,4]. High-entropy alloys are generally composed of five or more elements as the main element, and each element is between 5% and 35% (atomic percent). Although there are many principal elements in high-entropy alloys, the reported high-entropy alloys instead show a simple solid solution structure, exhibit both high strength and toughness in a large temperature range, high hardness and excellent wear resistance, good corrosion resistance and thermal stability and other excellent comprehensive properties [5,6,7]. Nutor et al. [8] reported that the AlCoNiV high-entropy alloy has high strength and toughness in the temperature range of 77 K to 723 K, and its specific yield strength reaches 150.2 MPa cm^3^/g. Dai et al. [9] showed that the addition of the Mo element could improve the Cr_2_O_3_/Cr(OH)_3_ ratio in the surface passivation film of FeCoCrNiMo_x_ high-entropy alloy in 3.5 wt.% NaCl solution and the corrosion resistance of the alloy were improved. Praveen et al. [10] reported that CoCrFeNi high-entropy alloy exhibited excellent thermal stability when exposed to 700 °C for 600 h with little change in hardness and grain size. The cavitation resistance of FeCoCrNi-based high-entropy alloys is 7.6 times that of 304 stainless steel [11]. In addition, the FeCoCrNi high-entropy alloy has good plasticity, with the elongation reaching 59% [12,13]. It is expected to become a new type of structural material in extreme marine environments.

Although high-entropy alloys have excellent properties, their engineering applications have been very limited so far. One of the reasons is the high cost of alloy preparation and processing. In general, the corrosion or wear of alloys starts from the surface. Surface coating technology plays a pivotal role in the corrosion and wear protection of engineering equipment [14,15,16]. The reported high-entropy alloy coatings also show unique structural and performance advantages. For example, Wang et al. [17] used laser cladding technology to prepare FeCoCrNi high-entropy alloy coatings on the Ti6Al4V alloy, and the wear resistance was improved by five times. Ye et al. [18] prepared an equimolar CrMnFeCoNi high-entropy alloy coating by laser cladding and the corrosion resistance of the coating in 3.5 wt.% NaCl solution and 0.5 M H_2_SO_4_ solution was better than that of the A36 steel. Wang et al. [19] prepared a (CoCrFeNi)_95_Nb_5_ high-entropy alloy coating on Q235 steel by laser cladding. When the laser energy density was 100 J·mm^−2^, the coating had the best corrosion resistance and highest hardness.

In terms of corrosion and wear protection of large-scale marine engineering equipment, laser cladding (LC) technology has a series of comprehensive advantages: such as relatively concentrated energy, fast heating and cooling rates, metallurgical bonding between coating and substrate, relatively small fusion, small heat-affected zone to the substrate, flexible operation, etc. [20,21,22,23,24] According to the literature reports [25], laser cladding high-entropy alloy coatings generally show typical dendrites and interdendritic structures. In order to elucidate the evolution of the microstructure of the coating and optimize its service performance, the methods generally adopted in the literature are to optimize the composition of the alloy by microalloying or to obtain a more excellent coating microstructure by optimizing the laser cladding process. Li et al. [26] obtained a fully dense FeNiCr cladding coating by optimizing the laser cladding process parameters. Wang et al. [19] reported the correlation between the laser scanning speed and the Laves phase content in (CoCrFeNi)_95_Nb_5_ coating. Zhang et al. [27] found that when FeCoNiCrCu coatings were doped with Si, Mn or Mo, the microstructure of the coatings was transformed from columnar and equiaxed grains to dendrites, and the microhardness was significantly improved. Up to now, the effect of microalloying on the microstructure of bulk high-entropy alloys has been widely reported, but there are few studies related to the influence of the microstructure and properties of high-entropy alloy coatings, and the research content is not systematic.

In this study, FeCoCrNi and FeCoCrNiMo_0.2_ high-entropy alloy powders were prepared by gas atomization. Then, high-entropy alloy coatings were prepared on the surface of 304 stainless steel by laser cladding technology. The influence of Mo on the microstructure and corrosion behavior of the FeCoCrNi high entropy alloy coating was studied.

## 2. Materials and Methods

### 2.1. Preparation of Powders by Gas Atomization

High-purity (>99.99%) bulk Fe, Co, Cr, Ni and Mo were selected as the raw materials. According to the nominal chemical composition of the high-entropy alloys shown in Table 1, FeCoCrNi and FeCoCrNiMo_0.2_ bulk high-entropy alloys were obtained by multiple smelting in an induction heating vacuum furnace. Then, the high entropy alloys were atomized with high-purity argon gas to obtain high-entropy alloy powders. The atomization pressure was 4 MPa. The high-entropy alloy powders were sieved, and FeCoCrNi (100–200 μm) and FeCoCrNiMo_0.2_ (100–200 μm) high-entropy alloy powders were selected as raw materials for the preparation of high-entropy alloy coatings.

### 2.2. Preparation of Laser Cladding Coatings

**Figure 1:**
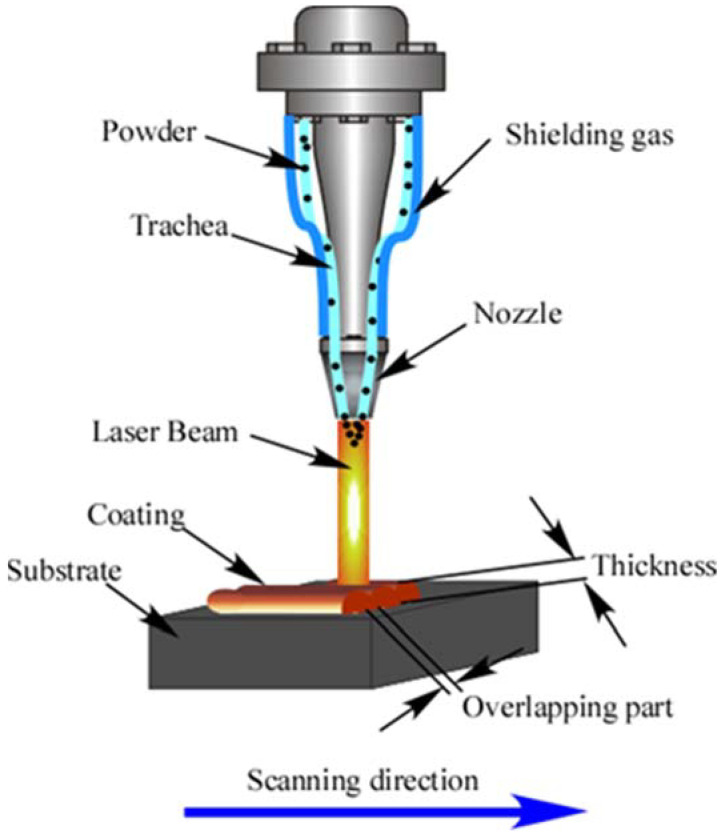
Schematic diagram of the preparation process of laser cladding coatings.

The substrate material selected in this study is SUS304 stainless steel, and its chemical composition is shown in Table 2. Samples of 100 mm × 100 mm × 8 mm are obtained from the stainless steel by using wire cutting technology. Before the preparation of the coating, in order to remove the oxide layer on the surface of the stainless steel, the surface is smoothed with sandpaper. The polished samples are cleaned with alcohol and then dried. The coating is prepared by using the JM-RB-3000 laser cladding equipment (Wuhan Jinmi Laser Technology Co., Ltd., Wuhan, China) with a coaxial powder feeding system. The preparation process is shown in Figure 1. High-purity argon is used as the protective gas to avoid oxidation of the molten pool. The preparation process parameters of the two high-entropy alloy coatings are shown in Table 3. The overlap ratio of adjacent cladding layers is selected to be 35%. The high-entropy alloy coating is obtained by single-layer multi-pass cladding.

### 2.3. Microstructure Characterization of Coatings

The FeCoCrNi and FeCoCrNiMo_0.2_ high-entropy alloy coatings were cut into 10 mm × 10 mm × 8 mm samples, which were ground with 240 #, 600 #, 800 #, 1000 # and 1200 # sandpapers, respectively, and finally polished. The phase identification of the samples was carried out by an AL-2700B X-ray diffractometer (Dandong Aolong Radiative Instrument Co., Ltd., Dandong, China). The test parameters are as follows: the target is Cu, the scanning range (2θ) is 10°~90°, the scanning speed is 5°/min, the tube voltage is 40 kV and the tube current is 30 mA. After etched with aqua regia solution, the cross-sectional morphology and chemical composition of the coatings were analyzed by a Phenom proX scanning electron microscope (Phenom-World, Eindhoven, The Netherlands) and energy dispersive spectrometer (EDS).

### 2.4. Electrochemical Measurements

Potentiodynamic polarization (PDP) and electrochemical impedance spectroscopy (EIS) analyses were carried out to investigate the corrosion behavior. A CHI660E electrochemical station (Shanghai Chenhua Instrument Co., Ltd., Shanghai, China) with a three-electrode cell system was used. All the measurements were conducted in 3.5 wt.% NaCl solution at room temperature (~25 °C). The test coating samples were welded with a copper wire and cold-mounted in epoxy. An exposed surface area of 1 cm^2^ served as a working surface. A platinum sheet and a saturated calomel electrode (SCE) were used as counter electrodes and reference, respectively.

Before testing, the samples are monitored for open circuit potential (OCP) for about 30 min. Then, the EIS measurements were performed in a frequency range from 100 kHz down to 10 mHz at OCP using a 10 mV signal amplitude. The PDP tests were started from −1 V and ended at 1.5 V versus OCP at a potential scan rate of 1 mV/s. All the electrochemical tests were performed at least three times. All the specimens were immersed in 3.5 wt.% NaCl solution for 24 h to form stable passive films. Then, X-ray photoelectron spectroscopy (XPS) with a monochromatic Al Kα X-ray radiation and a hemispherical electron analyzer was used to analyze the compositions of the passive films (Escalab 250xi, Thermo Fisher Scientific, Waltham, MA, USA). The XPS data were processed in commercial software Xpspeak version 4.1.

## 3. Results

### 3.1. Microstructure of Powder

**Figure 2:**
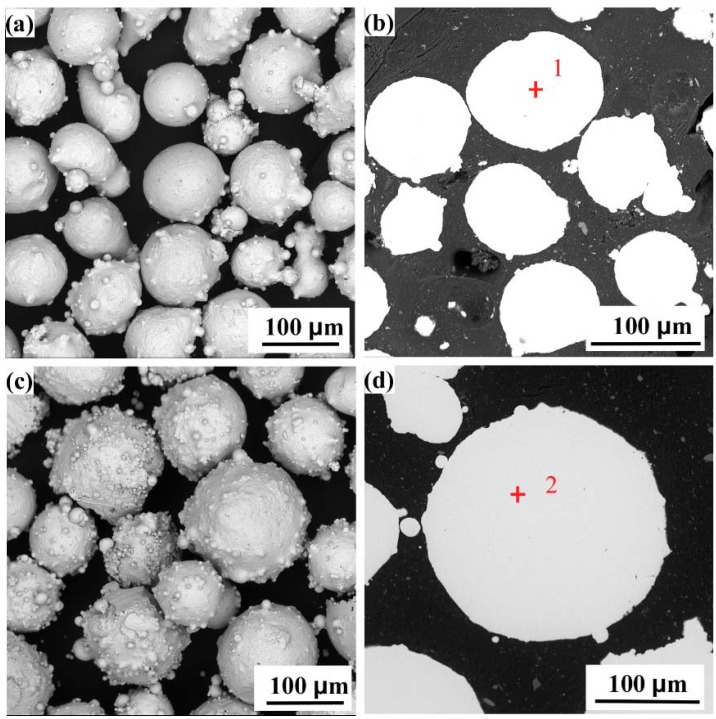
Surface and cross-sectional morphologies of high-entropy alloy powders. (**a**,**b**) FeCoCrNiMo_0.2_ powder; (**c**,**d**) FeCoCrNi powder.

Figure 2 shows the surface and cross-sectional morphologies of FeCoCrNi and FeCoCrNiMo_0.2_ high-entropy alloy powders prepared by gas atomization. It can be observed that the two powders are spherical. The internal structure of the powders is uniform, and there are no defects such as pores. Figure 2a,c shows that the two powders have satellite structures. This is because the faster the cooling rate of the smaller melt droplets, the easier it is to adhere to the surface of the large unsolidified droplets. The EDS analysis results of the positions, as marked as “1” and “2” in Figure 2b,d, are shown in Table 4. The results show that the powder composition prepared in this study is very close to the nominal composition in Table 1.

### 3.2. Phase Structure of Powder and Laser Cladding Coatings

**Figure 3:**
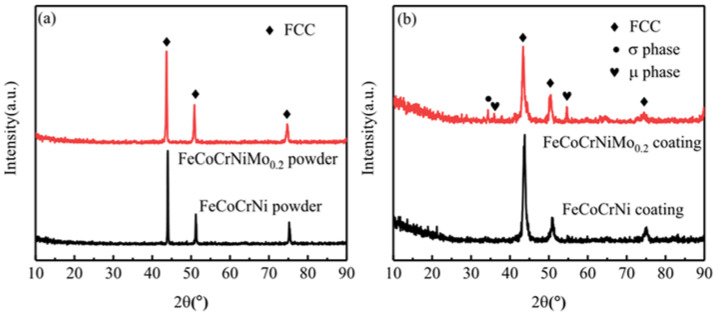
XRD patterns of powders and coatings. (**a**) XRD patterns of FeCoCrNi and FeCoCrNiMo_0.2_ powders; (**b**) XRD patterns of FeCoCrNi and FeCoCrNiMo_0.2_ coatings.

Figure 3 shows the XRD patterns of FeCoCrNi and FeCoCrNiMo_0.2_ spherical powders and their laser cladding coatings. It can be seen that both powders exhibit a typical single-phase FCC structure. The FeCoCrNi alloy coating is also a single-phase structure. However, a small amount of σ phase and μ phase appeared in FeCoCrNiMo_0.2_ alloy coating. The σ phase and μ phase are reported to exhibit tetragonal structure and hexagonal structure, respectively, and the μ phase may precipitate from the σ phase. Similar results were reported in the other literature [29,30].

The FeCoNiCrMo_0.3_ coating prepared by Dai et al. [31] by magnetron sputtering was mainly composed of the FCC phase with a small amount of σ phase. Liu et al. [13] reported that the σ phase appeared in the as-cast CoCrFeNiMo_0.3_ alloy, and the σ phase and μ phase appeared in the alloy after annealing. Niu et al. [30] prepared CoCrFeNiMo_x_ (x = 0, 0.2, 0.5, 0.8, 1) high-entropy alloys by arc melting and homogenization annealing. After annealing, the σ phase was found in the Mo_0.2_ alloy, and the σ phase and μ phase were found in the Mo_0.5_ alloy. The results show that the subsequent annealing promotes the formation of the σ phase in the Mo_0.2_ alloy. He et al. [32] proposed that the addition of elements with larger atomic sizes such as Mo or Nb increases lattice distortion, which may lead to phase separation of the CoCrFeNi alloy.

The overlap ratio of adjacent cladding layers in this study was 35%. During the preparation of the second cladding layer, part of the previous coating would remelt and solidify. The subsequent cladding layer performs multiple heat treatments on the previous coating, which may easily lead to the precipitation of the second phase. In this study, FeCoCrNiMo_0.2_ powder is FCC single-phase structure. The reason is that in the process of gas atomization, the cooling rate of powder particles is too large, and the alloy is in an unstable supersaturated solid solution state. However, a small amount of σ phase and μ phase appeared in FeCoCrNiMo_0.2_ coating because the FeCoCrNiMo_0.2_ high-entropy alloy coating was unstable, and phase separation may occur after heat treatment.

### 3.3. Microstructure of Laser Cladding High-Entropy Alloy Coatings

**Figure 4:**
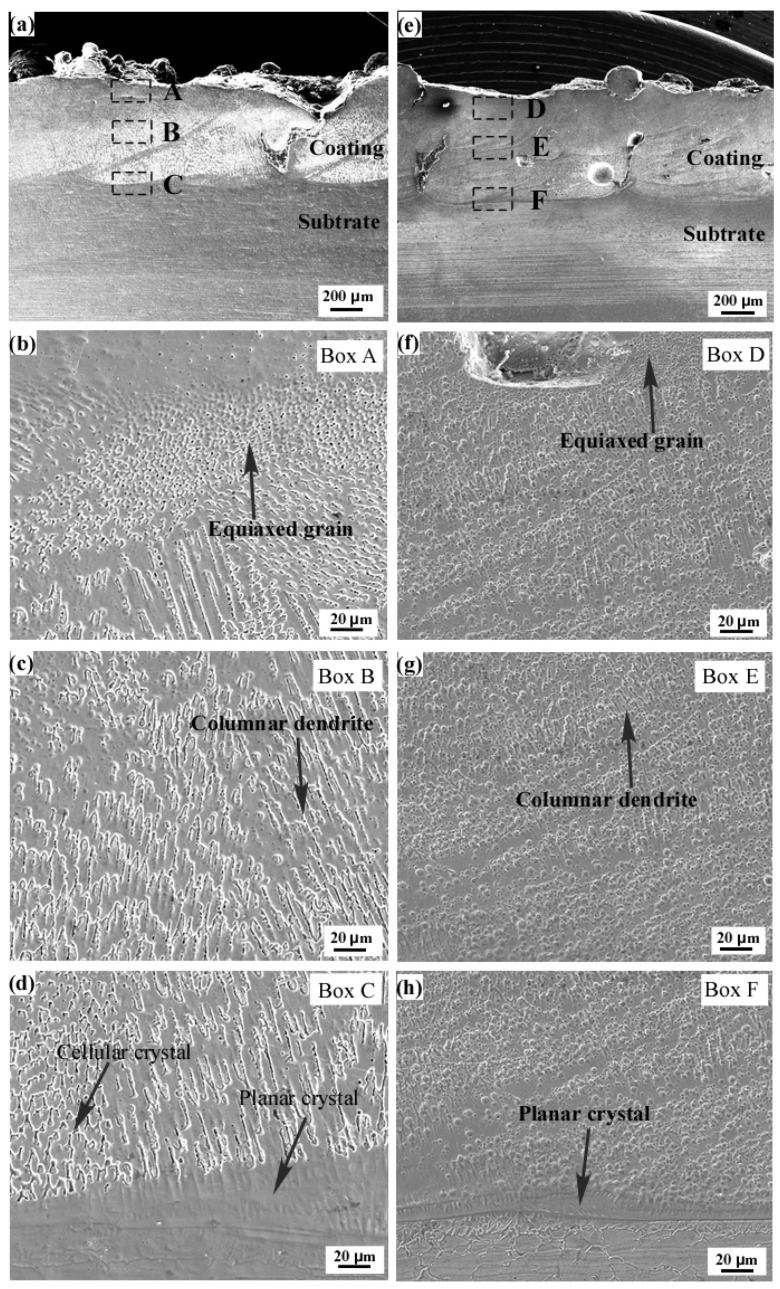
Cross-sectional morphologies of the coatings. (**a**–**d**) FeCoCrNiMo_0.2_ laser cladding coating; (**e**–**h**) FeCoCrNi laser cladding coating.

Figure 4a,e shows the cross-sectional microstructure of FeCoCrNiMo_0.2_ coating and FeCoCrNi coating, respectively. The results show that the microstructure of the coating is dense and uniform. There is a clear boundary between the multi-pass cladding layers with a small number of cracks and holes. The coating exhibits a good metallurgical bond with the SUS304 substrate. The top layer, the middle region and the bonding region with the substrate of the two coatings were selected to characterize the microstructure in detail, as shown in the regions A, B and C marked in Figure 4a and the D, E and F regions marked in Figure 4e. Figure 4b–d,f–h are the local microstructures of FeCoCrNiMo_0.2_ and FeCoCrNi coatings, respectively. The results show that the microstructures of the two coatings from the bonding area to the top layer are planar, columnar and equiaxed grains, respectively. Planar grains appear in the bonding area between the coating and the substrate because the temperature gradient is the largest and the grain growth rate is slow [33]. After the addition of the Mo element, the region of planar grains at the bottom of the coating becomes larger. Compared with the FeCoCrNi coating, the size of the dendrites in the middle of the FeCoCrNiMo_0.2_ coating increases significantly and shows obvious orientation characteristics. In addition, the size of dendrites on the top of the coating became smaller and gradually transformed into equiaxed grains, and the structure of the coating was uniform.

**Figure 5:**
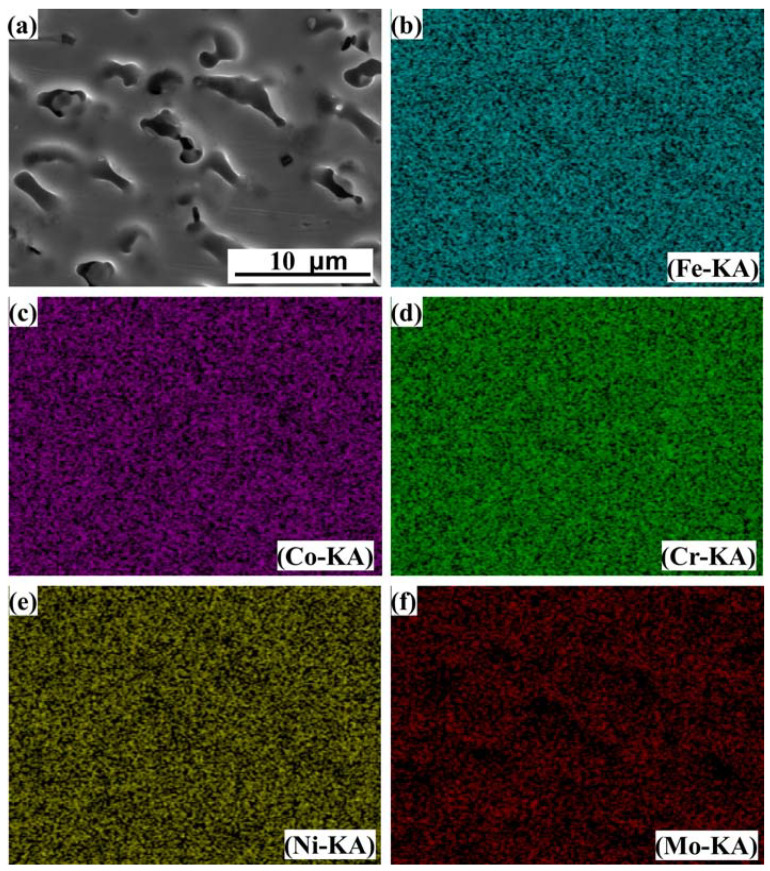
EDS analysis results of laser cladding FeCoCrNiMo_0.2_ coating. (**a**) SEM image; (**b**) Fe; (**c**) Co; (**d**) Cr; (**e**) Ni; (**f**) Mo.

**Figure 6:**
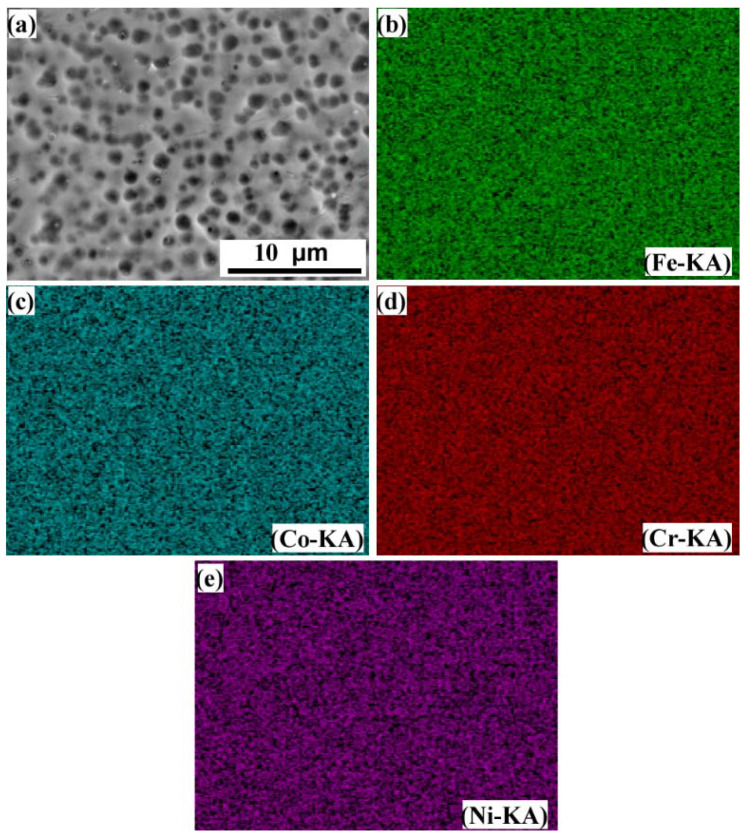
EDS analysis results of laser cladding FeCoCrNi coating. (**a**) SEM image; (**b**) Fe; (**c**) Co; (**d**) Cr; (**e**) Ni.

EDS analysis was performed on the B region annotated in Figure 4a and the E region annotated in Figure 4e, and the results are shown in Figure 5 and Figure 6, respectively. It can be seen from Figure 5 that Fe, Co, Cr and Ni elements are uniformly distributed in the dendrites and interdendritic regions in the FeCoCrNiMo_0.2_ coating, and the Mo element is enriched in the interdendritic regions. This may be related to the precipitation of the σ phase and μ phase. These phenomena are similar to the findings in the literature, such as the existence of (Cr, Mo)-enriched σ phase in FeCoCrNiMo_0.2_ coatings reported by Dai [31], and the existence of (Cr, Mo)-enriched σ phase and (Mo, Cr)-enriched μ phase in the as-cast CoCrFeNiMo_0.85_ alloy reported by Shun et al. [29]. It can be seen from Figure 6 that the distribution of each element in the FeCoCrNi coating is uniform, and there is no segregation phenomenon.

### 3.4. Corrosion Behavior of Laser Cladding High-Entropy Alloy Coatings

#### 3.4.1. Potentiodynamic Polarization Curves

**Figure 7:**
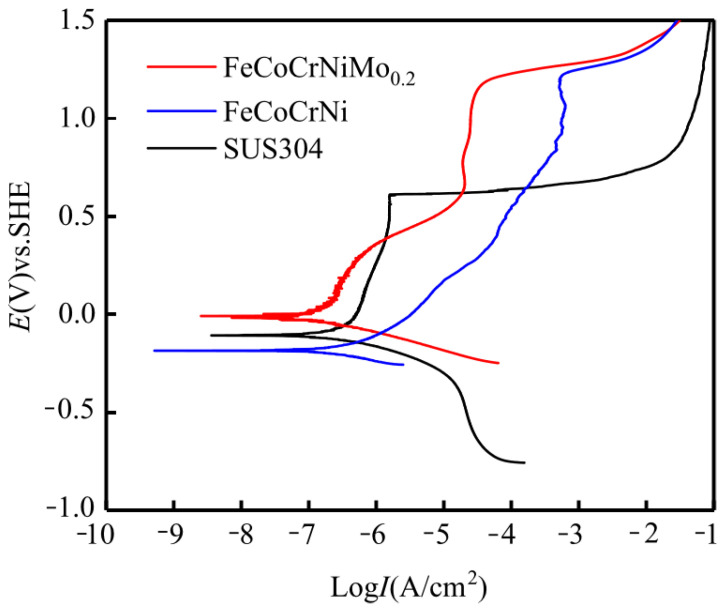
Potentiodynamic polarization curves of the laser cladding coatings and SUS304 stainless steel in 3.5 wt.% NaCl solution.

Figure 7 shows the potentiodynamic polarization curves of two high-entropy alloy coatings and 304 stainless steel in 3.5 wt.% NaCl solution. A series of kinetic parameters obtained from Figure 7 (*E*_corr_ is corrosion potential; *I*_corr_ is corrosion current density; *E*_br_ is breakdown potential) are shown in Table 5. The results show that the corrosion current density *I*_corr_ (0.94 × 10^−7^ A/cm^2^) of FeCoCrNiMo_0.2_ coating is lower than that of FeCoCrNi coating (1.64 × 10^−7^ A/cm^2^) and SUS304 stainless steel (2.10 × 10^−7^ A/cm^2^). The smaller the corrosion current density, the slower the corrosion rate of the coating.

The anodic polarization curves of FeCoCrNi and FeCoCrNiMo_0.2_ coatings both show a flat region where the current density increases slowly, indicating that the coatings enter the passivation region. The passivation range of FeCoCrNiMo_0.2_ coating is slightly wider than that of FeCoCrNi coating, which proves that the passivation film of FeCoCrNiMo_0.2_ coating is stable and exhibits good corrosion resistance. The breakdown potential means that the passivation film is destroyed after the potential value increases to a certain value [34]. The breakdown potential *E*_br_ of the FeCoCrNiMo_0.2_ (1.17 *V*_SHE_) and FeCoCrNi coating (1.20 *V*_SHE_) is significantly higher than that of the SUS304 stainless steel (0.60 *V*_SHE_), indicating that the two coatings have better corrosion resistance.

Shi et al. [35] reported the electrochemical corrosion properties of the Al_0.5_CoCrFeNi bulk high-entropy alloy under the same experimental conditions. It was found that with the increase of Al content, the volume fraction of the Cr-depleted phase increased, and the corrosion resistance decreased. As shown in Figure 4 and Figure 5, a small amount of (Cr, Mo)-enriched σ and (Mo, Cr)-enriched μ phases appeared in the FeCoCrNiMo_0.2_ coating in this study, which may reduce the corrosion resistance of the coating. The reason for this is that the appearance of σ-phase and μ-phase leads to the consumption of passivating elements such as Cr/Mo. In addition, galvanic corrosion may occur between the σ-phase, μ-phase and (Fe, Co, Ni, Cr) FCC solid solution phase to accelerate the corrosion process.

#### 3.4.2. Electrochemical Impedance Spectroscopy

**Figure 8:**
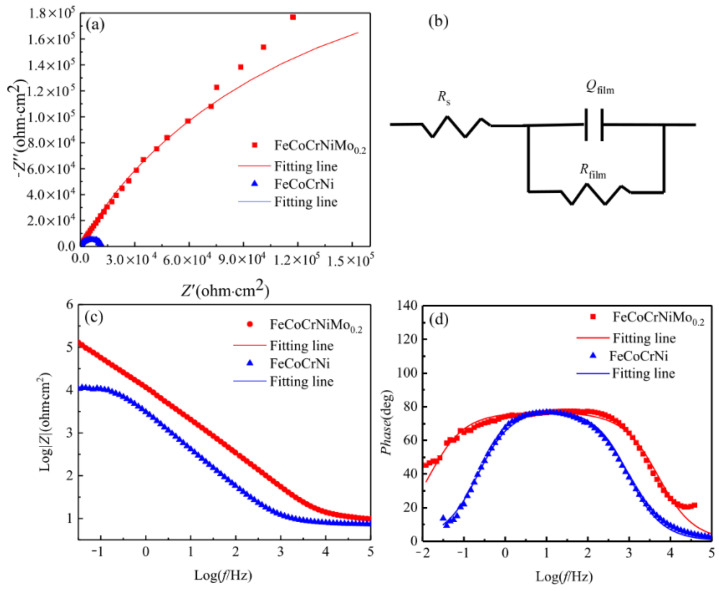
Electrochemical impedance spectrum of coatings. (**a**) Nyquist diagram; (**b**) schematic diagram of fitting circuit; (**c**,**d**) Bode diagram.

The EIS measurement results of the laser cladding FeCoCrNi and FeCoCrNiMo_0.2_ coatings under OCP conditions in 3.5 wt.% NaCl solution are shown in Figure 8. The Nyquist plots of all samples are incomplete semicircles (Figure 8a), which indicates that the corrosion process of the coating is controlled by the charge transfer process [36,37]. The semi-arc diameter for the FeCoCrNiMo_0.2_ coating is larger than that of the FeCoCrNi coating, indicating that a more protective passive film formed on the FeCoCrNiMo_0.2_ coating with high resistance to electrochemical dissolution. In the Bode plots of Figure 8c, the slope of the *logZ-logf* curve of each sample is close to -1, which means that the passive film formed on the coatings is pseudo-capacitive [38]. Only a single peak can be observed on the *Phase-log f* graph (Figure 8d), which implies one relaxation time constant [35]. According to the literature reports [19,39], an equivalent circuit *R*_s_*(Q*_film_*R*_film_*)*, including a pair of series-parallel circuits, is used to simulate EIS data, as shown in Figure 8b. In the EEC model, *R*_s_ represents the solution resistance, *R*_film_ corresponds to the charge transfer resistance of the electrode/electrolyte interface. Due to the heterogeneity of the electrode surface, the equivalent capacitance *Q*_film_ (constant phase angle element CPE) replaces “pure capacitance” C to simulate the capacitance of the electric double layer [40]. Fitted parameters are presented in Table 6.

As shown in Table 6, the Chi-squared(*χ*^2^) value is used to evaluate the quality of data fitting [14]. Its magnitude is 10^−3^, indicating that the fitting accuracy is high. The *R*_film_ value of FeCoCrNiMo_0.2_ coating is one order of magnitude higher than that of FeCoCrNi coating, indicating that the passive film has stable performance and low sensitivity to anions in solution. According to the literature [41,42], capacitance is closely related to the thickness and density of the passivation film. The capacitance *Q*_film_ value of FeCoCrNiMo_0.2_ coating is 2.18 × 10^−5^ Ω^−1^·cm^−2^·s^−n^, which is lower than that of FeCoCrNi coating (6.09 × 10^−5^ Ω^−1^·cm^−2^·s^−n^), indicating that the passivation film on the surface of the FeCoCrNiMo_0.2_ coating is thicker and denser. This result is consistent with the analysis results of potentiodynamic polarization curves in 3.5 wt.% NaCl solution.

#### 3.4.3. Surface Morphologies of Corroded Coatings

**Figure 9:**
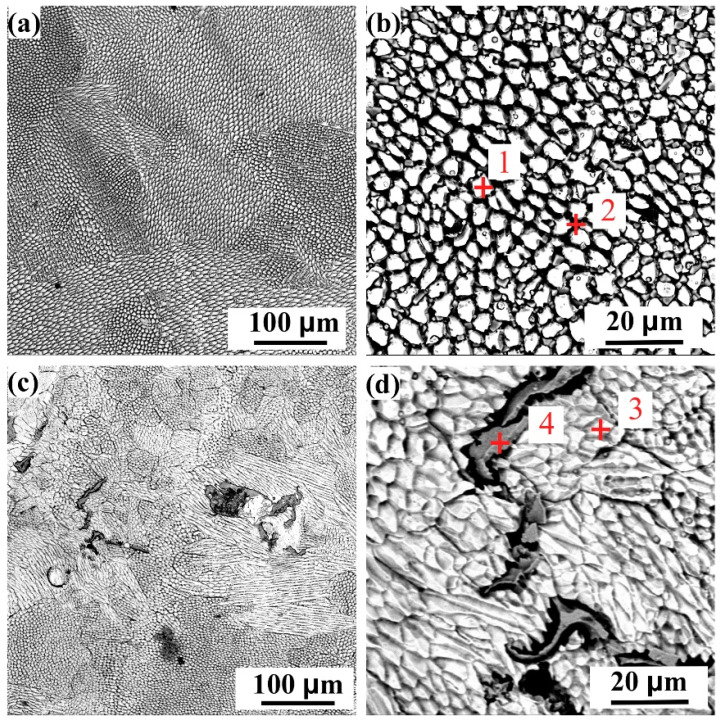
Surface morphologies of the corroded coatings. (**a**,**b**) FeCoCrNiMo_0.2_ coating; (**c**,**d**) FeCoCrNi coating.

Figure 9 shows the surface morphologies of FeCoCrNiMo_0.2_ and FeCoCrNi coatings after pitting corrosion. In general, corrosion occurs preferentially from grain boundaries. It can be seen from Figure 9 that both coatings suffered severe corrosion at the grain boundaries. It can be observed that the surface morphology of the corroded FeCoCrNiMo_0.2_ coating is uniform. There are many large corrosion areas and a small number of cracks on the surface of FeCoCrNi coating. These defects will facilitate the penetration of corrosive media into the coating. The corrosion products on the surfaces of the two coatingswere analyzed by EDS at the positions as marked as “1”, “2”, “3”and “4” in Figure 9b,d. The results are shown in Table 7. Cr and Mo are enriched in the black corrosion area on the surface of the FeCoCrNiMo_0.2_ coating. The corrosion products on the surface of FeCoCrNi coating are mainly oxides of Fe, Co, Ni and Cr. There is an enrichment of Cr in the flaky corrosion area.

#### 3.4.4. Passive Film Analysis

**Figure 10:**
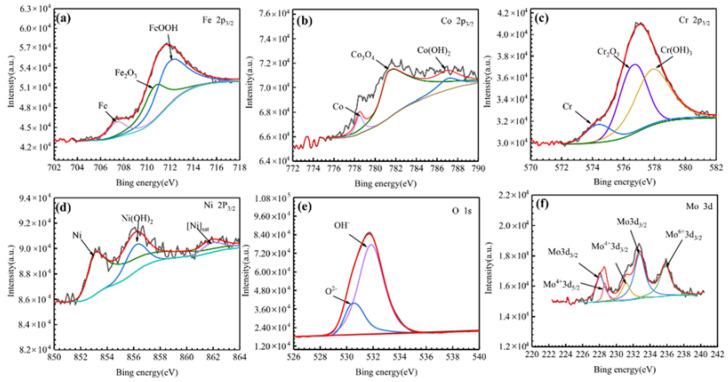
High resolution XPS spectra of the passive films formed on the FeCoCrNiMo_0.2_ coating in 3.5 wt.% NaCl solution. (**a**) Fe 2p_3/2_; (**b**) Co 2p_3/2_; (**c**) Cr 2p_3/2_; (**d**)Ni 2p_3/2_; (**e**) O 1s; (**f**) Mo 3d.

X-ray photoelectron spectroscopy (XPS) measurement was carried out to analyze the chemical composition information of the passivation films. Figure 10 shows the fitting results of the X-ray photoelectron spectrum of the passivation film formed on the surface of FeCoCrNiMo_0.2_ coating. The results showed that the passivation film on the FeCoCrNiMo_0.2_ coating is composed of hydroxides and oxides of Fe, Cr, Co, Ni and Mo. The literature studies have shown that [15,43] Mo^6+^ forms MoO_3_ in 3.5 wt.% NaCl solution, which can improve the stability of the passivation film and inhibit the occurrence of corrosion. Mo^4+^ can form MoO_2_ to hinder corrosive ions, thereby improving the corrosion resistance of the coating [44].

**Figure 11:**
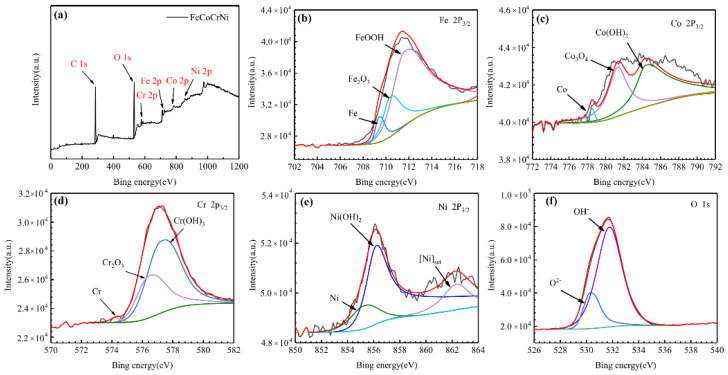
High resolution XPS spectra of the passive films formed on the FeCoCrNi coating in 3.5 wt.% NaCl solution. (**a**) survey spectrum; (**b**) Fe 2p_3/2_; (**c**) Co 2p_3/2_; (**d**) Cr 2p_3/2_; (**e**) Ni 2p_3/2_; (**f**) O 1s.

Figure 11 shows the fitting results of the X-ray photoelectron spectrum of the passivation film on the FeCoCrNi coating surface. According to Figure 11, the passivation film on the surface of FeCoCrNi coating consists of Fe, Cr hydroxides and oxides and a smaller amount of Co, Ni oxides and hydroxides. The fitting results of the above two coatings both show the spectral fitting results of Cr 2P_3/2_ [41,45], indicating that the passivation film is mainly composed of Cr, Cr_2_O_3_and Cr(OH)_3_. With the increase in passivation time during the corrosion process, Cr(OH)_3_ on the coating surface was hydrolyzed to Cr_2_O_3_, forming a stable Cr_2_O_3_ passivation film.

**Figure 12:**
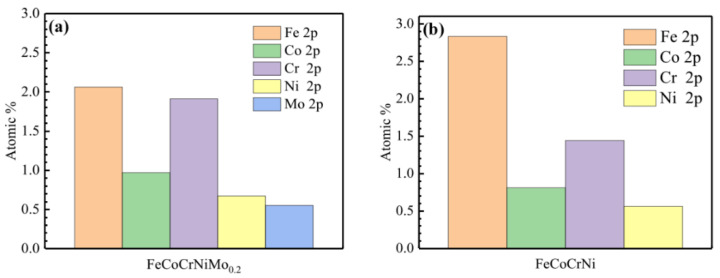
Cationic fractions in the passive films. (**a**) FeCoCrNiMo_0.2_ coating; (**b**) FeCoCrNi coating.

The fractions of cationic and metallic species of Fe, Co, Cr, Ni and Mo in passivation film formed on the FeCoCrNi and FeCoCrNiMo_0.2_ coatings were plotted in Figure 12. The results show that the atomic ratio of Cr/Fe in the passive film formed on the surface of FeCoCrNi coating is 0.46. After adding 4.76 at.% Mo to FeCoCrNi alloy, the atomic ratio of Cr/Fe in the passivation film on the FeCoCrNiMo_0.2_ coating increased to 0.93. In addition, there is a relatively high content of Mo in the passivation film on the FeCoCrNiMo_0.2_ coating, which improves the compactness and stability of the passivation film. It was reported in the literature that the addition of Mo promotes the deprotonation of Cr(OH)_3_, thereby increasing the Cr_2_O_3_ content in the passivation film [46]. Cr(OH)_3_ contains more bound water, has a loose structure and has poor protective properties. Cr_2_O_3_ is a key component in the passivation film and plays a decisive role in the corrosion resistance of the coating. Generally, the higher the Cr content in the passivation film, the better the corrosion resistance of the coating.

## 4. Discussion

### 4.1. Influence of Mo Doping on the Microstructure of Powders and Coatings

It can be seen from Figure 2 that the FeCoCrNi and FeCoCrNiMo_0.2_ powders are spherical. The powder surface is a satellite structure. The internal structure of the powder is uniform, and there are no defects such as pores. Figure 3 shows that the FeCoCrNiMo_0.2_ powder still maintains the single-phase FCC structure. However, the σ phase and μ phase other than the FCC phase appeared in FeCoCrNiMo_0.2_ coating [30]. This is mainly because, in the process of gas atomization, the powder particles are small, and the cooling rate is fast, resulting in the FeCoCrNiMo_0.2_ powder being in a supersaturated solid solution state. During the coating preparation process, the FeCoCrNiMo_0.2_ coating in the supersaturated solid solution state has undergone multiple remelting and continuous heat treatments, resulting in the phase separation of the alloy. Figure 4 shows that the microstructures of the two coatings from the bonding area to the top layer are mainly planar, columnar and equiaxed grains. The addition of Mo leads to a significant increase in the size of dendrites in the FeCoCrNiMo_0.2_ coating and shows obvious orientation characteristics.

### 4.2. Influence of Mo Doping on the Corrosion Resistance of Coatings

**Figure 13:**
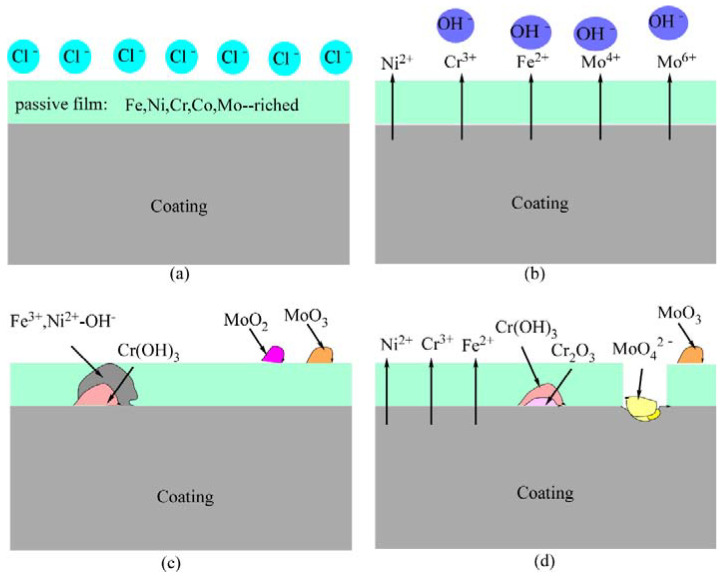
Schematic diagram of electrochemical corrosion process of FeCoCrNiMo_0.2_ coating in 3.5 wt.% NaCl solution. (**a**) passivation film formation process; (**b**) galvanic corrosion process; (**c**) The composition of the passivation film; (**d**) dissolution process of passivation film.

Compared with FeCoCrNi coating, FeCoCrNiMo_0.2_ coating has higher corrosion potential and lower corrosion current in 3.5 wt.% NaCl solution. The passivation film on the FeCoCrNiMo_0.2_ coating contains high content of Cr and Mo, showing excellent corrosion resistance. The electrochemical corrosion process of the FeCoCrNiMo_0.2_ coating is shown in Figure 13. The XPS analysis results show that the Mo^6+^ and Mo^4+^ in the passivation film of FeCoCrNiMo_0.2_ coating can improve the corrosion resistance of the coating. In the passivation region, passivation film rich in Fe, Ni, Cr, Co and Mo elements is formed on the surface of the coating (Figure 13a). Galvanic corrosion occurs due to the potential difference between the grains and grain boundaries (Figure 13b). The ionized metal ions combine with OH^−^ in solution, and Cr(OH)_3_ preferentially nucleates and grows. Due to the faster diffusion rate of Fe and Ni, Fe^3+^ and Ni^2+^-OH^−^ are formed in the outer layer of Cr(OH)_3_ [39]. Mo^4+^ can form MoO_2_ to hinder corrosive ions, and Mo^6+^ in NaCl solution to form MoO_3_ exists on the surface of the passivation film, thereby preventing the occurrence of corrosion [44]. According to the literature reports [43,47], MoO_3_ is mainly concentrated in the outer layer of the passivation film, while MoO_2_ is mainly formed at 1 nm of the passivation film (Figure 13c). As the potential increases, Fe^3+^ and Ni^2+^-OH^−^ begin to dissolve, while Cr(OH)_3_ in the inner layer begins to form dense Cr_2_O_3._ MoO_2_ is further oxidized to form MoO_4_^2−^ intermetallic compounds, which accumulate at pitting corrosion and effectively prevent corrosion. MoO_3_ still exists on the surface of the passivation film, which can significantly improve the stability of the passivation film (Figure 13d). The protective ability of the passivation film is the key to the corrosion resistance of the alloy [48], so the corrosion resistance of the coating is better after adding Mo.

Many studies [49,50] also confirmed that dense oxides such as Cr_2_O_3_ and MoO_3_ have good pitting resistance in chlorine-containing environments. Wei et al. [51] reported that in 3.5 wt.% NaCl solution, MoO_4_^2−^ intermetallic compounds formed on the surface of Ni_2_CrFeMo_0.2_ high-entropy alloys, which gathered at places where pitting pits preferentially occurred. Zheng et al. [52] investigated the inhibitory effect of MoO_4_^2−^ on hydrogen permeation of 2205 duplex stainless steel. MoO_4_^2−^ is adsorbed on the metal surface, resulting in the reduction in the hydrogen permeation current density, reducing the pitting corrosion on the metal surface, and improving the corrosion resistance of stainless steel. Shang et al. [53] showed that the addition of Mo improved the corrosion resistance of CoCrFeNiW_1−x_Mo_x_ (*x* = 0, 0.5) high-entropy alloy coatings in 3.5 wt.% NaCl solution.

## 5. Conclusions

(1) Both FeCoCrNi and FeCoCrNiMo_0.2_ high-entropy alloy powders prepared by gas atomization are single-phase FCC structures. Due to the remelting and multiple heat treatments during the preparation of the laser cladding coating, a small amount of σ phase and μ phase appeared in the FeCoCrNiMo_0.2_ coating;
(2) The microstructure of the two coatings from the bonding area to the top layer is planar, columnar and equiaxed grains, respectively. After adding the Mo element, the region of the plane grain in FeCoCrNiMo_0.2_ coating becomes larger. The size of dendrites in the middle of the FeCoCrNiMo_0.2_ coating increased significantly and showed obvious orientation characteristics;
(3) The corrosion current densities of FeCoCrNi and FeCoCrNiMo_0.2_ coatings are 0.78 and 0.45 times that of 304 stainless steel, respectively. This indicates that both coatings exhibit excellent corrosion resistance and passivation performance in 3.5 wt.% NaCl solution. Compared with FeCoCrNi coating, the passivation film on the surface of FeCoCrNiMo_0.2_ coating contains high content of Cr and Mo oxides. The addition of Mo element enhances the compactness and pitting resistance of the passivation film.

## Figures and Tables

**Table 1:** Nominal chemical compositions of FeCoCrNi and FeCoCrNiMo_0.2_ high-entropy alloys (at.%).

	Co	Cr	Ni	Fe	Mo
FeCoCrNi	25	25	25	25	--
FeCoCrNiMo_0.2_	23.81	23.81	23.81	23.81	4.76

**Table 2:** Chemical composition of 304 stainless steel (wt.%) [28].

Cr	Ni	Mn	Si	P	S	C	Fe
18~20	8~11	2	1	0.045	0.03	0.08	Bal

**Table 3:** Laser cladding process parameters.

Sample	Laser Power (W)	Spot Diameter (mm)	Overlap Rate (%)	Scanning Velocity (mm/min)	Feeding Rate (g/min)
FeCoCrNi	2100	2	35	400	16
FeCoCrNiMo_0.2_	2100	2	35	300	18

**Table 4:** EDS analysis results at the markers in Figure 2 (at.%).

	Fe	Co	Cr	Ni	Mo	O
1	20.89	20.47	21.76	20.15	4.93	11.80
2	23.62	23.61	21.74	22.81	--	8.22

**Table 5:** Electrochemical parameters obtained from potentiodynamic polarization curves.

	*E*_corr_ (*V*_SHE_)	*I*_corr_ (A·cm^−2^)	*E*_br_ (*V*_SHE_)
FeCoCrNiMo_0.2_	−0.01	0.94 × 10^−7^	1.17
FeCoCrNi	−0.19	1.64 × 10^−7^	1.20
SUS304	−0.11	2.10 × 10^−7^	0.60
Al_0.5_CoCrFeNi [35]	−0.015	2.52 × 10^−7^	--

**Table 6:** Electrochemical parameters obtained from the potentiodynamic polarization curves.

	*R*_s_ (Ω·cm^2^)	*Q*_film_ (Ω^−1^·cm^−2^·s^−n^)	*R*_film_ (Ω·cm^2^)	*N*	*χ* ^2^
FeCoCrNiMo_0.2_	8.64	2.18 × 10^−5^	5.59 × 10^5^	0.77	6.45 × 10^−3^
FeCoCrNi	7.78	6.09 × 10^−5^	1.25 × 10^4^	0.88	1.94 × 10^−3^

**Table 7:** EDS analysis results of corrosion products formed on the corroded coatings at the marked positions as shown in Figure 9.

	Fe	Ni	Co	O	Mo	Cr
1	20.81	19.89	18.28	22.66	3.41	14.94
2	21.63	15.76	14.42	27.16	2.89	18.15
3	22.79	22.16	21.44	15.20	--	18.42
4	6.75	7.06	7.50	31.74	--	37.70

## Data Availability

Not applicable.
